# Synthesis and preclinical evaluation of novel ^18^F-labeled Glu-urea-Glu-based PSMA inhibitors for prostate cancer imaging: a comparison with ^18^F-DCFPyl and ^18^F-PSMA-1007

**DOI:** 10.1186/s13550-018-0382-8

**Published:** 2018-04-12

**Authors:** Stephanie Robu, Alexander Schmidt, Matthias Eiber, Margret Schottelius, Thomas Günther, Behrooz Hooshyar Yousefi, Markus Schwaiger, Hans-Jürgen Wester

**Affiliations:** 1 Chair of Pharmaceutical Radiochemistry, Technical University Munich, Walther-Meissner-Strasse 3, 85748 Garching, Germany; 2 Department of Nuclear Medicine, Klinikum rechts der Isar, Technical University Munich, Ismaningerstr. 22, 81675 Munich, Germany

**Keywords:** PSMA, ^18^F-labeled EuE-based inhibitors, ^18^F-DCFPyl, ^18^F-PSMA-1007, PET prostate Cancer imaging

## Abstract

**Background:**

Due to its high and consistent expression in prostate cancer (PCa), the prostate-specific membrane antigen (PSMA) represents an ideal target for molecular imaging and targeted therapy using highly specific radiolabeled PSMA ligands. To address the continuously growing clinical demand for ^18^F-labeled PSMA-probes, we developed two novel Glu-urea-Glu-(EuE)-based inhibitors, EuE-k-^18^F-FBOA (1) and EuE-k-β-a-^18^F-FPyl (2), both with optimized linker structure and different ^18^F-labeled aromatic moieties. The inhibitors were evaluated in a comparative preclinical study with ^18^F-DCFPyl and ^18^F-PSMA-1007.

**Results:**

Radiolabeling procedures allowed preparation of (1) and (2) with high radiochemical yields (67 ± 7 and 53 ± 7%, d.c.) and purity (> 98%). When compared with ^18^F-DCFPyl (IC_50_ = 12.3 ± 1.2 nM) and ^18^F-PSMA-1007 (IC_50_ = 4.2 ± 0.5 nM), both metabolically stable EuE-based ligands showed commensurable or higher PSMA affinity (IC_50_ = 4.2 ± 0.4 nM (1), IC_50_ = 1.1 ± 0.2 nM (2)). Moreover, 1.4- and 2.7-fold higher internalization rates were observed for (1) and (2), respectively, resulting in markedly enhanced tumor accumulation in LNCaP-tumor-bearing mice ((1) 12.7 ± 2.0% IA/g, (2) 13.0° ± 1.0% IA/g vs. 7.3 ± 1.0% IA/g (^18^F-DCFPyl), 7.1 ± 1.5% IA/g (^18^F-PSMA-1007), 1 h p.i.). In contrast to (1), (2) showed higher kidney accumulation and delayed clearance kinetics. Due to the high hydrophilicity of both compounds, almost no unspecific uptake in non-target tissue was observed. In contrast, due to the less hydrophilic character (logP = − 1.6) and high plasma protein binding (98%), ^18^F-PSMA-1007 showed uptake in non-target tissue and predominantly hepatobiliary excretion, whereas, ^18^F-DCFPyl exhibited pharmacokinetics quite similar to those obtained with (1) and (2).

**Conclusion:**

Both ^18^F-labeled EuE-based PSMA ligands showed excellent in vitro and in vivo PSMA-targeting characteristics. The substantially higher tumor accumulation in mice compared to recently introduced ^18^F-PSMA-1007 and ^18^F-DCFPyl suggests their high value for preclinical studies investigating the effects on PSMA-expression. In contrast to (2), (1) seems to be more promising for further investigation, due to the more reliable ^18^F-labeling procedure, the faster clearance kinetics with comparable high tumor uptake, resulting therefore in better high-contrast microPET imaging as early as 1 h p.i.

**Electronic supplementary material:**

The online version of this article (10.1186/s13550-018-0382-8) contains supplementary material, which is available to authorized users.

## Background

During the last several years, the prostate-specific membrane antigen (PSMA) and corresponding radiolabeled inhibitors have become one of the most extensively investigated target/tracer pair for molecular imaging and radioligand therapy of prostate cancer (PCa). Due to readily availability of ^68^Ge/^68^Ga-generators, strong emphasis has been placed on the development and optimization of ^68^Ga-labeled PET probes for clinical imaging of PCa. However, the use of ^68^Ga for labeling of PSMA inhibitors has severe limitations. Based on the small generator sizes, the overall activity that can be produced in a single batch production is quite low and only sufficient in optimal conditions for three to four patients (maximum of 1500 MBq). In addition, due to short half-life of only 68 min, ^68^Ga-PSMA ligands have to be produced in big centers several times a day to cope with high clinical need. Due to the unique radionuclide characteristics of ^18^F (*t*_1/2_ = 109.7 min, *E*_ß+_ = 0.63 MeV) and its corresponding advantages for clinical PET imaging combined with large-scale production by means of even small cyclotrons, several groups have focused on the development of ^18^F-labeled PSMA inhibitors for PCa imaging [1–6]. One of the first ^18^F-labeled PSMA ligand was ^18^F-DCFBC, demonstrating the ability for detection of high-grade primary PCa and metastatic lesions [4]. However, ^18^F-DCFBC possessed some features that could be improved through further refinements in the chemical structure. Especially, the high plasma protein binding of the tracer, which results in slow clearance kinetics and high blood pool activity can interfere with the detection of lower avidity or smaller tumor lesions [7, 8].

The second-generation inhibitor ^18^F-DCFPyl, developed by the same group [2], showed five times higher PSMA affinity, improved tumor uptake, and rapid plasma clearance, resulting in higher tumor-to-blood and tumor-to-background ratios and lower accumulation in the liver compared to ^18^F-DCFBC. However, a considerable kidney and salivary gland uptake was observed [9].

Recently, ^18^F-PSMA-1007, a novel ^18^F-labeled tracer based on the DKFZ-617-scaffold [1, 10], was reported. First human studies exhibit excellent sensitivity of ^18^F-PSMA-1007 for the detection of small lymph node metastases. In contrast to the renal clearance of ^18^F-PSMA-1007 in animal studies, predominant hepatobiliary excretion with reduced urinary uptake was observed in this first series of patients. Overall, a major disadvantage is the slow tracer kinetic of ^18^F-PSMA-1007, resulting in favorable tumor-to-background ratios and an increased tumor uptake up to 50% at late imaging time points (3 h p.i.) [11].

**Fig. 1:**
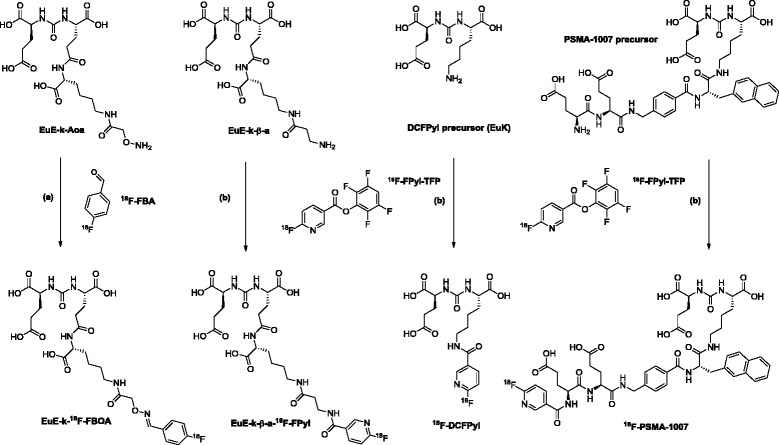
Synthesis and chemical structures of the ^18^F-labeled EuE-based inhibitors (EuE-k-^18^F-FBOA and EuE-k-β-a-^18^F-FPyl), ^18^F-DCFPyl and ^18^F-PSMA-1007 [1, 8, 15]. **a** Reaction conditions: ^18^F-FBA, EuE-k-AoA (3.3 μmol), 15 min at 60 °C [MeCN/water (1/1), (*v*/v), acidified with TFA to pH 2.5]. **b** Reaction conditions: ^18^F-FPyl-TFP, EuE-k-β-a/EuK/PSMA-1007 precursor (3.9 μmol), 15 min at 60 °C, NaHCO_3_ (5.5 mg) [MeCN/water; 2/1, (*v*/*v*)]

In this study, we aimed towards the development of novel ^18^F-labeled PSMA inhibitors, exploiting optimized ^18^F-labeling strategies and the increasing experience concerning the structural requirements for optimal ligand binding to further improve the in vitro and in vivo PSMA-targeting characteristics in comparison to the currently available ^18^F-labeled compounds. Up to date, the development of PSMA inhibitors has been mainly based on the Lys-urea-Glu (KuE) core as binding motif [1, 2, 12]. However, in a preliminary study, the design of PSMA inhibitors containing a EuE (Glu-urea-Glu) binding motif was described [13]. A direct comparison of both urea-based binding motifs was demonstrated by Hillier et al., who evaluated different ^99m^Tc-labeled PSMA inhibitors, based on either a KuE- or a EuE-binding motif with related linker structures and chelator moieties [14]. In vitro and in vivo data successfully demonstrated, the beneficial influence of a free carboxylic group in the linker region, i.e., by introduction of an amino acid to the EuE-based binding motif. This structural difference had pronounced effect on the hydrophilicity of the ligand and resulted in enhanced PSMA affinity, higher internalization efficiency, higher tumor accumulation, and favorable clearance kinetics [14]. Therefore, we designed two alternative EuE-based ligands suitable to be labeled with either chemo-selective oxime ligation (as a generally applicable ^18^F-labeling strategy for peptidic ligands) or established acylation chemistry with ^18^F-FPyl-TFP. Both ^18^F-labeled analogs, named EuE-k-^18^F-FBOA and EuE-k-ß-a-^18^F-FPyl, were subsequently evaluated in terms of PSMA affinity, internalization in LNCaP PCa cells, metabolic stability, micro PET imaging, and in vivo biodistribution. The recently introduced ligands, ^18^F-DCFPyl [2, 9] and ^18^F-PSMA-1007 [1, 15], were included in this evaluation process to allow a direct comparison of all four tracers (Fig. 1).

## Methods

### Chemical synthesis and radiolabeling

A detailed description of the chemical synthesis and radiolabeling of all compounds is provided in the supporting information.

### Lipophilicity and plasma protein binding

To a solution of ^18^F-labeled peptide (0.5–1.0 MBq) in 0.5 mL PBS (pH 7.4), 0.5 mL of octanol was added (*n* = 6). The vials were vortexed vigorously for 3 min. To achieve efficient phase separation, vials were centrifuged for 5 min at 6000*g* in a Biofuge 15 (Heraeus Sepatech, Osterode, Germany). Aliquots (100 μL) of the aqueous and the octanol phase were collected and the radioactivity concentrations in the respective samples were quantified using a γ-counter. The Log P_O/PBS_ values were calculated from the means of *n* = 6 separate determinations.

Plasma protein binding of the tracers was determined using an analytical Chiralpak human serum albumin (HSA) column (50 × 3 mm, 5 μm) according to a previously published protocol with minor changes [16].

### In vitro evaluation

#### Cell culture

PSMA overexpressing LNCaP cells (CLS: 300265) were cultured in DMEM/Nutrition Mix F-12 with Glutamax-I (1:1) (Invitrogen, Life Technologies, Darmstadt, Germany) supplemented with 10% FCS and were maintained at 37 °C in a 5% CO_2_/humidified air atmosphere. For IC_50_ determination, approximately 150,000 cells/well were seeded on 24-well plates 1 day prior to the experiment. For internalization studies, 125,000 cells/well were seeded in PLL-coated 24-well plates. For cell counting, a Countesse automated cell counter (Invitrogen, Carlsbad, USA) was used.

#### Determination of IC_50_ and internalization studies

PSMA affinity and internalization kinetics of the ^18^F-labeled compounds were determined according to a previously published protocol [17]. A detailed description is provided online in the supporting information.

Competitive binding experiments (*IC*_*50*_) were carried out using PSMA-expressing LNCaP cells and ([^125^I]I-BA)KuE as standard radioligand. Internalization kinetics of the ^18^F-labeled derivatives were also performed using PSMA-expressing LNCaP cells and ([^125^I]I-BA)KuE (0.2 nM) as an internal reference. Data were corrected for non-specific internalization in the presence of 100 μM 2-phosphonomethyl pentanedioic acid (PMPA) and normalized to the specific internalization observed for the radioiodinated reference compound assayed in a parallel experiment. Data represent means ± SD (*n* = 3).

### Metabolite analyses

Approximately 60–70 MBq of the ^18^F-labeled inhibitors were injected into the tail vein of severe combined immunodeficiency (SCID) mice. The animals were sacrificed 1 h p.i., blood and urine were collected, and kidneys were dissected and after freezing with liquid nitrogen, homogenized with a ball mill and extracted with 1 mL PBS containing 200 nmol PMPA. After centrifugation (15,000*g*) and ultrafiltration, the extracts were analyzed by reversed phase high-performance liquid chromatography (RP-HPLC). Blood samples were centrifuged to separate the plasma from the blood cells. Additionally, plasma proteins were removed by precipitation with acetonitrile (10 min, on ice), subsequent centrifugation and ultrafiltration. The blood extracts and the urine samples were analyzed using RP-HPLC. For RP-HPLC, a Nucleosil 100 C18 (5 μm, 125 × 4.0) column and different HPLC-systems were used (EuE-based inhibitors: flow rate: 2 mL/min; Gradient: 0–30% B in 20 min; HPLC-System A (see Additional file 1); ^18^F-PSMA-1007: flow rate: 1.5 mL/min; Gradient: 5–55% B in 10 min; HPLC-System B (see Additional file 1); solvent A: 0.1% trifluoroacetic acid (TFA) in water, solvent B: 0.1% TFA in MeCN).

### In vivo evaluation

#### General

All animal experiments were conducted in accordance with the German Animal Welfare Act (Deutsches Tierschutzgesetz, approval no. 55.2-1-54-2532-71-13).

The LNCaP cells were attached from the surface of the culture flask using Trypsin/EDTA (0.05 and 0.02%) in PBS, centrifuged, and resuspended in culture medium. After cell counting, the cells were again centrifuged and resuspended 1/1 in serum-free culture medium and Matrigel (BD Biosciences, Germany). Concentrations of the cell suspension were approximately 1 × 10^7^ cells/200 μL.

#### Animal model

To induce LNCaP tumor growth, male CB-17 SCID mice (6–8 weeks, Charles River Laboratories, Sulzfeld, Germany) were inoculated subcutaneously onto the right shoulder with LNCaP cells (approximately 1 × 10^7^ cells/200 μL DMEM/Nutrition Mix F-12 with Glutamax-I (1:1) medium and Matrigel (BD Biosciences, Germany) (1:1)). After 3–5 weeks, tumor size reached 4–8 mm in diameter, and the animals were used for in vivo studies.

#### Small-animal PET imaging

In vivo imaging studies were performed using a Siemens Inveon small-animal PET scanner. Mice were injected into the tail vein with app. 0.9–1 MBq of the respective ^18^F-labeled compound under isoflurane anesthesia. For competition experiments, 2-PMPA (1 μmol = 226 μg/mouse) was coinjected. Dynamic images were recorded for 1.5 h after on-bed injection. Reconstruction of the images was carried out using three-dimensional ordered-subsets expectation maximum algorithm without scanner and attenuation correction. Data analysis was carried out using Inveon Workplace software.

#### Biodistribution

About 0.8–1 MBq of the ^18^F-labeled tracer were injected into the tail vein of the mice (*n* = 8) under isoflurane anesthesia. Animals were sacrificed at 1 and 2 h p.i.; the organs of interest were dissected, and the activity in the weighed tissues samples was quantified using a γ-counter.

## Results

### Chemical synthesis

The *t*Bu-protected PSMA binding motifs (O*t*Bu)EuE(O*t*Bu)_2_ and (O*t*Bu)KuE(O*t*Bu)_2_ were synthesized in 84 and 92% yield via three steps by solution phase synthesis as previously described [17]. The DCFPyl precursor (Fig. 1) was obtained by direct deprotection of (O*t*Bu)KuE(O*t*Bu)_2_ using TFA (68% yield after preparative RP-HPLC). The EuE-based- and PSMA-1007-precursors (Fig. 1, Additional file 1: Figure S1A) were prepared via solid phase peptide synthesis. The respective labeling precursors were cleaved from the resin using TFA, with concomitant removal of acid labile protecting groups (54–63% yield after RP-HPLC purification). The corresponding cold reference ligands EuE-k-β-a-FPyl, F-DCFPyl, and F-PSMA-1007 were subsequently prepared by direct conjugation of the respective precursors with 2,3,5,6-tetrafluorophenyl 6-fluoronicotinate under basic conditions and were obtained in 82, 75, and 87% yield after RP-HPLC purification, respectively. EuE-k-FBOA was obtained via chemo-selective oxime ligation of EuE-k-Aoa with 4-fluorobenzaldehyde under acidic aqueous conditions in 87% yield after RP-HPLC purification (Additional file 1: Figure S1A). The identity of all final products was confirmed by ESI-MS.

### ^18^F-radiolabeling

The radiosynthesis of the prosthetic groups ^18^F-FBA and ^18^F-FPyl-TFP was performed according to a previously published approach (Additional file 1: Figure S1B) [18, 19]. ^18^F-fluoride was directly eluted with an alcoholic solution (EtOH/MeOH) of the corresponding quaternary ammonium precursor salt, followed by heating of the resulting ^18^F-fluoride salt in a suitable solvent (MeCN/^*t*^BuOH/EtOH, DMSO) without any additives or base. The ^18^F-labeled prosthetic groups ^18^F-FBA and ^18^F-FPyl-TFP were isolated via SPE extraction procedures [18, 19], which allowed quantitative separation from unreacted labeling precursor. ^18^F-FBA was synthesized within 35 min with high RCY (70–84%, d.c.) and high RCP (> 94%, *n* = 4), ^18^F-FPyl-TFP in 53–78% RCY (*n* = 12, d.c.) with > 75% RCP within 35 min. Coupling of ^18^F-FPyl-TFP was performed without further purification.

One-step oxime ligation of ^18^F-FBA and EuE-k-Aoa (3.2 μmol) generally resulted in conjugation yields > 98% (*n* = 4) within 15 min at 60 °C. RP-HPLC isolation of EuE-k-^18^F-FBOA afforded the ^18^F-labeled product in high RCY (56–75%, d.c.; based on ^18^F-FBA and RCP (> 98%) in a synthesis time of app. 50 min (Fig. 1)).

The acylation of the EuE-k-β-a and DCFPyl precursors (3.9 μmol, respectively) with ^18^F-FPyl-TFP was highly efficient, with conjugation yields of about 93% and 90%, respectively. In contrast, only very low conjugation yields of about 21% were achieved during the synthesis of ^18^F-PSMA-1007 (Fig. 1). After RP-HPLC purification, RCYs of 45–64% (EuE-k-β-a-^18^F-FPyl), 45% (^18^F-DCFPyl), and 10% (^18^F-PSMA-1007) (d.c.) in a preparation time of about 40–50 min for all ligands were achieved, respectively. RCPs of the purified ^18^F-labeled peptides (n.c.a.) were > 98%.

### In vitro studies

**Fig. 2:**
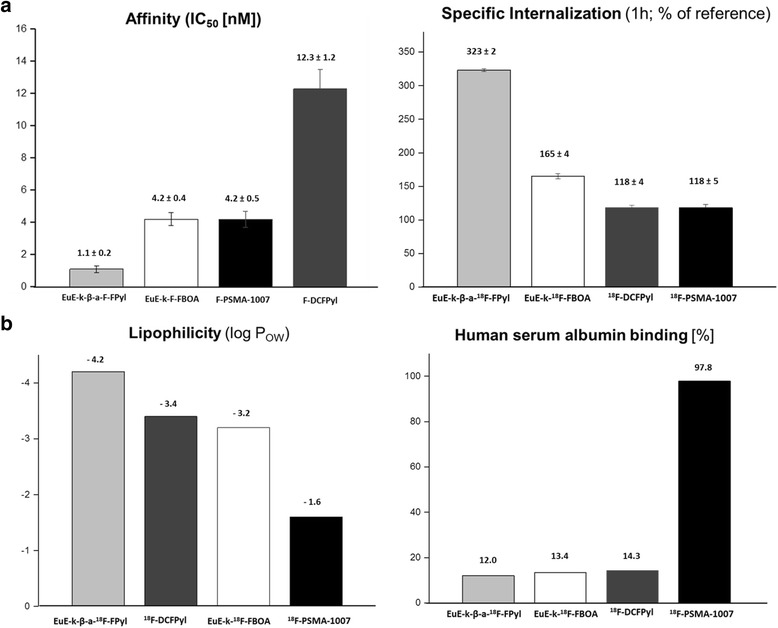
**a** PSMA affinities of the cold EuE-based inhibitors, F-DCFPyl and F-PSMA-1007. Internalization of the ^18^F-labeled EuE-based PSMA inhibitors, EuE-k-^18^F-FBOA and EuE-k-β-a-^18^F-FPyl in comparison to ^18^F-DCFPyl and ^18^F-PSMA-1007. **b** Lipophilicity and HSA-binding of EuE-k-^18^F-FBOA, EuE-k-β-a-^18^F-FPyl, ^18^F-DCFPyl, and ^18^F-PSMA-1007. *Specific internalization of the reference compound ((^125^I)I-BA)KuE assayed in a parallel experiment and used for data normalization

Binding affinities (*IC*_*50*_) of the cold reference ligands EuE-k-FBOA, EuE-k-β-a-FPyl, F-DCFPyl, and F-PSMA-1007 were determined in a competitive binding assay using LNCaP human PCa cells and ([^125^I]I-BA)KuE as radioligand (0.2 nM) [20]. Inhibition curves of the respective PSMA inhibitors are shown in the Additional file 1: Figure S2 and data are summarized in Fig. 2a.

EuE-k-FBOA and F-PSMA-1007 exhibited nearly identical and high affinities towards PSMA, whereas F-DCFPyl showed a threefold lower binding affinity. The highest PSMA affinity was observed for EuE-k-β-a-FPyl with an IC_50_ 4- to 12-fold higher than that determined for EuE-k-FBOA, F-PSMA-1007, and F-DCFPyl, respectively.

In addition, marked differences in the internalization efficiency of the ^18^F-labeled EuE-based inhibitors in comparison to the two reference compounds ^18^F-DCFPyl and ^18^F-PSMA-1007 were observed (Fig. 2a). Compared to EuE-k-^18^F-FBOA, EuE-k-β-a-^18^F-FPyl showed substantially (twofold) enhanced internalization into LNCaP cells and exhibited the highest internalization efficiency of all compounds investigated in this study. Despite significant different PSMA affinities, ^18^F-DCFPyl and ^18^F-PSMA-1007 showed identical internalization rates, but markedly lower overall internalization into PSMA-expressing cells when compared to the ^18^F-labeled EuE-based compounds (Fig. 2a, Additional file 1: Figure S3).

### Lipophilicity and plasma protein binding

The lipophilicities and human serum albumin (HSA) binding of all ^18^F-labeled compounds are summarized in Fig. 2b. Although the logP values of EuE-k-^18^F-FBOA and ^18^F-DCFPyl were nearly identical (− 3.2 and − 3.4, respectively) and the logP of EuE-k-β-a-^18^F-FPyl was even lower by an order of magnitude (− 4.2), all three compounds exhibited similar, low plasma protein binding in the range of 12–14%. Due to its significantly higher lipophilicity (− 1.6), a markedly enhanced plasma protein binding of 98% was found for ^18^F-PSMA-1007.

### Metabolite analysis

The metabolic stability of the ^18^F-labeled EuE-based compounds EuE-k-^18^F-FBOA and EuE-k-β-a-^18^F-FPyl, as well as of ^18^F-PSMA-1007 was investigated in CD-1 mice (1 h p.i.). The metabolic stability of ^18^F-DCFPyl in mice has been reported elsewhere [21]. No in vivo degradation of both EuE-based tracers was observed in blood, urine, and kidneys (100% intact tracer) at 1 h p.i., while ^18^F-PSMA-1007 showed an slight in vivo degradation to a more hydrophilic metabolite, amounting to 2% of the total activity in the urine and 4% in the kidneys at 1 h p.i. (Additional file 1: Figure S4).

### Small-animal PET studies

**Fig. 3:**
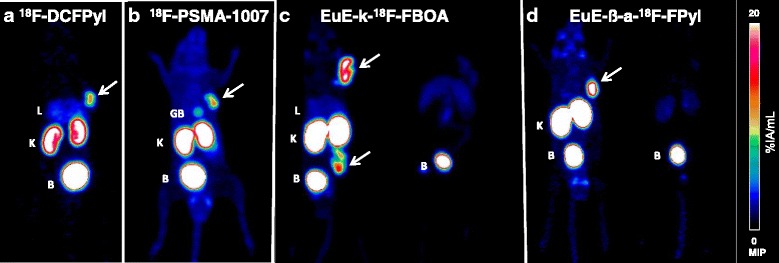
Maximum intensity projections (MIP) of micro PET scans. Summation image (60–90 min p.i.; 0% to 20% IA/mL) of LNCaP-tumor-bearing SCID mice after injection of approximately 0.9–1 MBq of **a** ^18^F-DCFPyl; **b** ^18^F-PSMA-1007; **c** left: EuE-k-^18^F-FBOA, right: EuE-k-^18^F-FBOA + blocking with 8 mg/kg PMPA; **d** left: EuE-k-β-a-^18^F-FPyl, EuE-k-β-a-^18^F-FPyl + blocking with 8 mg/kg (PMPA). LNCaP tumors are indicated with arrows (please note that the animal shown in panel **c** bears LNCaP tumors at the right shoulder and on the flank of the hind leg). Organs are marked as B = bladder, GB = gallbladder, K = kidney, L = liver

Dynamic micro PET imaging (0–90 min p.i.) of EuE-k-^18^F-FBOA, EuE-k-β-a-^18^F-FPyl, ^18^F-DCFPyl, and ^18^F-PSMA-1007 was carried out in LNCaP-tumor-bearing SCID mice (Fig. 3). A comparison of the pharmacokinetics of the four radiofluorinated inhibitors as measured by micro PET revealed a slightly enhanced tracer uptake of ^18^F-DCFPyl in the liver compared to the other ^18^F-labeled analogs. Increased tracer accumulation in the gallbladder was observed for ^18^F-PSMA-1007, probably due to a slight shift from renal to hepatobiliary excretion. In addition, ^18^F-PSMA-1007 displayed a delayed blood clearance (Additional file 1: Figure S5), due to its high-pronounced plasma protein binding, which results in an increased background activity in micro PET images.

In contrast, the EuE-based inhibitors and ^18^F-DCFPyl displayed rapid blood clearance (Additional file 1: Figure S5) with low unspecific whole body uptake and predominant renal clearance. Although all three inhibitors exhibited similar low plasma protein binding, EuE-k-β-a-^18^F-FPyl showed slightly increased background activity in micro PET. Interestingly, compared to the reference ligands ^18^F-PSMA-1007 and ^18^F-DCFPyl, both ^18^F-labeled EuE-based inhibitors, revealed enhanced uptake in the salivary and lacrimal glands.

Due to the enhanced internalization efficiency of the EuE-based inhibitors, markedly higher uptake in tumor lesions was observed compared to ^18^F-DCFPyl and ^18^F-PSMA-1007. Tracer uptake of EuE-k-^18^F-FBOA and EuE-k-β-a-^18^F-FPyl into tumor and kidneys is specific and PSMA-mediated, as demonstrated by blocking experiments with PMPA (Fig. 3).

### Biodistribution

**Fig. 4:**
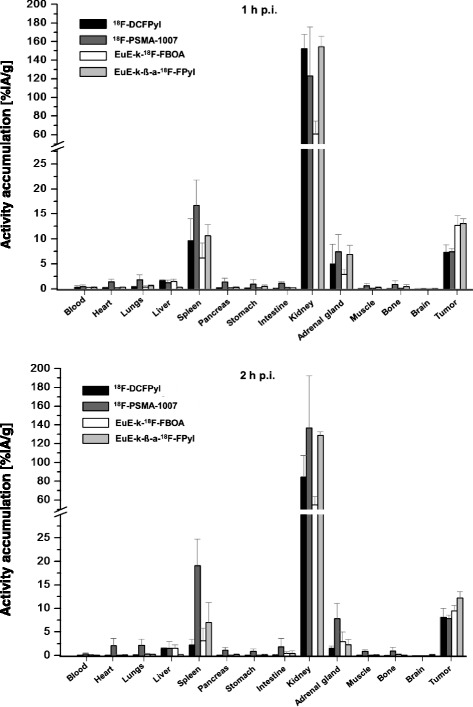
Biodistribution of EuE-k-^18^F-FBOA and EuE-k-β-a-^18^F-FPyl in comparison to ^18^F-DCFPyl and ^18^F-PSMA-1007 in LNCaP-tumor-bearing SCID mice at 1 and 2 h p.i.. Data are given in % IA/g are means ± SD (*n* = 4 animals per group and time point)

**Fig. 5:**
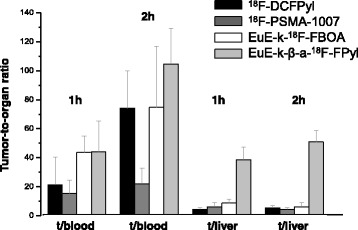
Tumor-to-organ ratios for EuE-k-^18^F-FBOA and EuE-k-β-a-^18^F-FPyl in comparison to ^18^F-DCFPyl and ^18^F-PSMA-1007 in LNCaP-tumor-bearing SCID mice at 1 and 2 h p.i

A comparison of the in vivo biodistribution of the EuE-based inhibitors EuE-k-^18^F-FBOA and EuE-k-β-a-^18^F-FPyl and of the EuK analogs ^18^F-DCFPyl and ^18^F-PSMA-1007 in LNCaP-tumor-bearing SCID mice (1 h (*n* = 4) and 2 h p.i. (*n* = 4)) is shown in Fig. 4. EuE-k-^18^F-FBOA, EuE-k-β-a-^18^F-FPyl, and ^18^F-DCFPyl exhibited similar pharmacokinetics with fast renal excretion and low activity levels in non-target tissues. Interestingly, while all other compounds show low, but detectable liver accumulation, nearly no uptake for EuE-k-β-a-^18^F-FPyl was observed, which resulted in high tumor-to-liver ratios (Fig. 5) for this tracer. In contrast, ^18^F-PSMA-1007 showed markedly slower pharmacokinetics with increased and delayed delivery of the tracer in non-target tissue, the gastrointestinal tract and PSMA-mediated organs, like the spleen, kidneys, and adrenal glands over time (2 h p.i.). The high plasma protein binding and therefore delayed blood pool clearance of ^18^F-PSMA-1007 leads to low tumor-to-blood and tumor-to-spleen ratios, especially 2 h p.i. (Fig. 5; Additional file 1: Figure S5).

All compounds generally show high but variable tracer uptake in organs with physiological expression of the murine PSMA-variant, like the kidneys, the adrenal glands, and the spleen [22, 23]. However, of the compounds investigated, EuE-k-^18^F-FBOA showed the lowest accumulation in these organs, resulting in high tumor-to-organ, and especially high tumor-to-kidney ratios (Fig. 5).

In comparison to the other two tracers, the EuE-based inhibitors EuE-k-^18^F-FBOA and EuE-k-β-a-^18^F-FPyl showed the highest tumor uptake at 1 and 2 h p.i.

## Discussion

The present study was focused on the development of novel ^18^F-labeled EuE-based PSMA inhibitors with optimized PSMA-targeting characteristics and favorable pharmacokinetics. To ensure a valid assessment of the new tracers, two recently introduced tracers, ^18^F-PSMA-1007 and ^18^F-DCFPyl, were coevaluated.

For efficient radiolabeling of both structurally related EuE-based ligands, the preparation of the ^18^F-labeled synthons ^18^F-FBA and ^18^F-FPyl-TFP was carried out using a recently introduced approach allowing for the direct conversion of an onium salt precursor into a ^18^F-labeled compound without the need of time-consuming azeotropic drying steps and addition of a base or other ingredients [18, 19].

Radiolabeling of both prosthetic groups was achieved with high RCYs comparable to those described in literature [18, 19]. However, in comparison to the oxime ligation with the ^18^F-labeled benzaldehyde ^18^F-FBA, the acylation approach revealed lower RCYs and RCP, most probably due to the hydrolysis of the labeled synthon and slower overall reaction kinetics. When compared to the high labeling efficiencies for ^18^F-DCFPyl and EuE-k-β-a-^18^F-FPyl, the conjugation efficiency of ^18^F-PSMA-1007 was found to be significantly decreased, resulting in markedly lower overall RCYs. Cardinale et al. explained the low conversation rate with the formation of an inner salt of the terminal glutamic acid and the amino group that might reduce the nucleophilicity of the amino group [1]. In contrast, the synthesis of EuE-k-^18^F-FBOA via oxime ligation with ^18^F-FBA resulted in good RCYs with less amount of precursor peptide needed. Moreover, nearly no side-product formation was observed. Therefore, we assume that the final HPLC purification might be replaceable by a SPE extraction in an automated production of EuE-k-^18^F-FBOA. Furthermore, due to ease of labeling, this approach should be well suited for automated radiosynthesis in clinical routine.

Regarding the ligand design, we focused on the improvement of the structural requirements for favorable in vivo and in vitro PSMA-targeting characteristics. As expected, all tracers show high affinity towards PSMA, although the affinity of F-DCFPyl was found to be significantly lower, and more comparable to those of ^68^Ga-HBED-CC and ^68^Ga-PSMA-I&T [24, 25].

Apart from a low nanomolar receptor affinity, the extent of peptide internalization is another, potentially even more important factor for efficient targeted tracer uptake. A study by Liu et al. has indicated that the internalization efficiency of a PSMA-inhibitor complex depends on the extent of PSMA conformational changes, resulting from different inhibition modes for PSMA ligands [26]. The binding of a ligand can either support or inhibit the interaction between the cytoplasmic tail of PSMA with clathrin and the clathrin adaptor protein-2 complex and thus alter the internalization efficiency [26, 27]. However, reports about structural features of PSMA inhibitors, which influence the internalization potency and to what extent, are scarce. A moderate correlation between increasing lipophilicity and higher cellular uptake of an inhibitor has been reported recently [28, 29]. However, in this study, the most hydrophilic compound EuE-k-β-a-^18^F-FPyl revealed markedly enhanced internalization compared to all compounds investigated.

Additionally, based on our unreported findings and on recently published data, there seems to be no direct correlation between PSMA affinity and internalization efficiency of an inhibitor [28, 29]. This assumption is consistent with our findings in which ^18^F-DCFPyl and ^18^F-PSMA-1007 showed identical internalization efficiency, despite their IC_50_ values being approximately twofold apart. Yet in contrast, the enhanced affinity of the EuE-based inhibitors was associated with 1.4-fold and 2.7-fold higher internalization rates in PSMA-expressing cells in comparison to the reference ligands. Therefore, further studies are needed to address these questions thoroughly.

In addition, the hydrophilicity (logP) and the extend of plasma protein binding significantly contributes to the performance and dominates the in vivo pharmacokinetics of a given radiopharmaceutical. An ideal PSMA-targeted PET tracer show a delayed urinary excretion with a slight shift towards hepatobiliary clearance, which is especially important for the detection of primary PCa and the early pattern of lymph node metastasis. Whereas EuE-k-^18^F-FBOA and ^18^F-DCFPyl fulfill these criteria (logP − 2…− 3), EuE-k-β-a-^18^F-FPyl exhibits a lower lipophilicity (− 4.2) and ^18^F-PSMA-1007 a higher lipophilicity (− 1.6), resulting in 98% plasma protein binding and thus the slowest excretion kinetics for ^18^F-PSMA-1007 as demonstrated in microPET imaging and biodistribution studies (Figs. 3 and 4; Additional file 1: Figure S5). In addition, metabolic studies of ^18^F-PSMA-1007 showed irreducible degradation in the urine (2%) and kidneys (4%), probably based on the intrinsic susceptibility of L-amino acid peptides towards degradation by endopeptidases (Additional file 1: Figure S4) [17].

As hypothesized, both EuE-based ligands showed straightforward clearance kinetics in micro PET images and biodistribution studies in LNCaP-xenograft-bearing mice. In addition to the fast renal clearance of these inhibitors, especially for EuE-k-^18^F-FBOA, almost no background activity or tracer accumulation in non-target tissue was observed (Figs. 3 and 4). Due to its high hydrophilicity and therefore predominant renal excretion, exclusively, EuE-k-β-a-^18^F-FPyl displayed no uptake in the liver. As anticipated, along with high plasma protein binding and long blood circulation, ^18^F-PSMA-1007 displayed a delayed blood clearance (Additional file 1: Figure S5) and non-specific accumulation in non-target tissue, like the heart, lungs, pancreas, stomach, intestine, muscle, and bone, which increases within 2 h p.i. (Fig. 4). Thus, later imaging time points may be required for high-contrast PCa imaging using ^18^F-PSMA-1007, which is in accordance with observations made in initial patient studies [10].

In terms of similarity, all ligands showed high but variable accumulation in murine PSMA-expressing organs, like the kidneys, the adrenal glands, and the spleen [22, 23]. The observed variability of respective tracer accumulation in these organs does not directly correlate with the determined PSMA affinity and accumulation in human LNCaP xenografts of the compounds investigated. These observations may be explained by considerable differences in affinities of the respective inhibitors for PSMA expressed on human (xenograft) tumors and murine PSMA expressed on mouse tissues.

Particularly, the uptake of ^18^F-PSMA-1007 in the spleen was significantly increased compared to the other inhibitors (Fig. 4). This variable uptake of PSMA-targeted radioligands in mouse spleen was already observed by us and others [13, 30–32]. Although PSMA (GCPII)-expression in mouse spleen is documented on the mRNA-level [23], it is not detectable on the protein level [22]. Nevertheless, blockable tracer uptake in mouse spleen has been observed for a multitude of small urea-based PSMA inhibitors, albeit at very variable levels (from 0.6% IA/g [13] to 47% IA/g [31] at 1 h p.i.), which does not correlate with the relative PSMA affinities of the compounds investigated. Our own data have already hinted towards a strong dependence of splenic tracer uptake on the respective mouse strain used [31], whereas tracer uptake in human LNCaP xenografts accurately reflects the respective expression density of human PSMA and thus allows valid comparisons [13, 25, 30, 32]. Therefore, blockable uptake of a given radiolabeled PSMA inhibitor in the mouse spleen, as well as other murine PSMA-expressing organs is a qualitative indicator for successful PSMA-targeting, but cannot provide quantitative and relative information on its PSMA-targeting performance in humans.

Interestingly, EuE-k-^18^F-FBOA and ^18^F-DCFPyl revealed significantly lower accumulation in the kidneys with faster renal clearance compared to EuE-k-β-a-^18^F-FPyl and ^18^F-PSMA-1007, which showed even higher accumulation after 2 h p.i. (Fig. 4). For ^18^F-PSMA-1007, this effect may be linked to the delayed delivery of the tracer caused by the higher activity levels in the blood pool (Additional file 1: Figure S5). For EuE-k-β-a-^18^F-FPyl, redistribution effects may contribute to this effect.

Since tracer internalization is a key factor for the efficiency of tumor uptake and retention, the higher internalization of EuE-k-^18^F-FBOA and EuE-k-β-a-^18^F-FPyl and the enhanced PSMA-targeting characteristics compared to ^18^F-DCFPyl and ^18^F-PSMA-1007 correlates with the approximately 50% enhanced tumor accumulation in micro PET imaging and biodistribution studies (12.7 ± 2.0% IA/g, 13.0 ± 1.0% IA/g, 1 h p.i. vs 7.3 ± 1.0% IA/g (^18^F-DCFPyl), 7.1 ± 1.5% IA/g (^18^F-PSMA-1007), 1 h p.i.) in LNCaP-tumor-xenografts (Figs. 3 and 4). Additionally, EuE-k-β-a-^18^F-FPyl revealed higher tumor retention 2 h p.i.. The better tumor-to-liver ratios for EuE-k-β-a-^18^F-FPyl were occasioned by the enhanced hydrophilicity, whereas EuE-k-^18^F-FBOA revealed better tumor-to-kidney ratios compared to the reference ligands, explainable by the low accumulation and fast renal clearance leading to high-contrast PET imaging (Figs. 3 and 5).

**Fig. 6:**
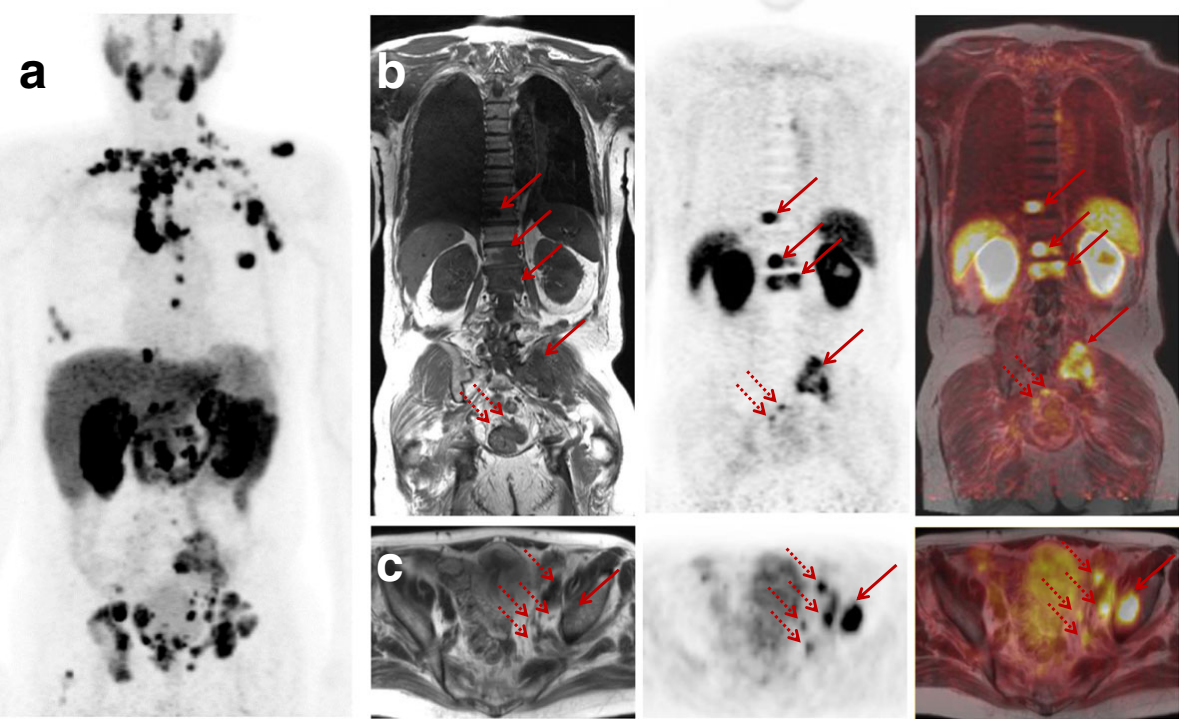
PET/MR imaging in a mCRPC patient (PSA 392 ng/ml) using EuE-k-^18^F-FBOA (1) demonstrates intensive PSMA-ligand uptake in multiple bone (arrows) and tiny subcentimeter lymph node (dotted arrows) metastases. Maximum intensity projection (**a**) shows distribution of disease. Sets **b** and **c** shows coronal and axial MR (T1 and T2 haste), PET and fused images with arrows and dotted arrows representing bone and lymph node metastases, respectively

Based on the promising preclinical results obtained for EuE-k-^18^F-FBOA (1) a 79-year-old mCRPC patient (PSA 392 ng/ml) was imaged under compassionate use. The agent was applied in compliance with The German Medicinal Products Act, Arzneimittelgesetz (AMG) §13 2b, and in accordance with the responsible regulatory body (Government of Oberbayern). The patient underwent PET/MR imaging 62 min after injection of 158 MBq of EuE-k-^18^F-FBOA (Fig. 6).

Images show the typical biodistribution of PSMA ligands with minimal blood pool retention. Based on the high hydrophilicity and low plasma protein binding of EuE-k-^18^F-FBOA, the rapid renal washout and fast blood clearance allowed high-contrast PCa PET imaging at 1 h p.i.. EuE-k-^18^F-FBOA revealed high uptake in the kidneys (SUV_mean_ 14.0), the salivary glands (SUV_mean_ 5.8), and the spleen (SUV_mean_ 6.1) and moderate uptake in the liver (SUV_mean_ 6.4), respectively. Figure 6 demonstrates intensive PSMA-ligand uptake in multiple bone and lymph node metastases with mean SUV_mean_ 14.7 (range 9.2–19) and mean SUV_max_ 21.6 (range 15.4–28.2). Notably, even tiny subcentimeter lymph node metastases (dotted arrows) showed intense uptake of (1) and were easily detectable. These results indicate high potential of EuE-k-^18^F-FBOA (1) for the detection of metastatic PCa even at early imaging time points.

## Conclusion

The ^18^F-labeled EuE-based PSMA ligands EuE-k-^18^F-FBOA and EuE-k-β-a-^18^F-FPyl showed excellent PSMA-affinities, pronounced hydrophilicity, low plasma protein binding, low unspecific uptake, and significantly higher tumor accumulation in mice than those obtained with the two recently introduced PSMA PET tracers, ^18^F-PSMA-1007 and ^18^F-DCFPyl. In addition, the preclinical comparison of the four ^18^F-labeled radiopharmaceuticals revealed significant different in vivo behaviors that might have an impact of the relative performance of these tracers in human studies. Based on high and persistent accumulation in targeted tissue, faster renal clearance of EuE-k-^18^F-FBOA resulted in better high-contrast microPET imaging and higher tumor-to-organ ratios at early time points of 1 h p.i. compared to EuE-k-β-a-^18^F-FPyl. Therefore, we expect EuE-k-^18^F-FBOA to be more promising for further clinical investigations, additionally, due to its reliable radiolabeling procedure facilitating the suitable transfer for automatization in clinical routine.

## Additional file

Additional file 1: Supporting information contains the description of the chemical synthesis and radiolabeling of all compounds investigated in this study, the methods and results for the determination of the PSMA binding affinities (IC_50_) and internalization studies, the metabolite analyses and the time-activity curves for the blood pool derived from dynamic small-animal PET. (PDF 911kb)

## Abbreviations

% IA/g: Percentage of injected activity per gram
% IA/mL: Percentage of injected activity per unit volume
^18^F-FBA: ^18^F-fluorobenzaldehyde
^18^F-FPyl-TFP: 2,3,5,6-Tetrafluorophenylester-6-^18^F-fluoronicotinate
AMG: Arzneimittelgesetz
DMEM: Dulbecco’s modified Eagle’s medium
EuE: Glu-urea-Glu
FCS: Fetal calf sera
HSA: Human serum albumin
IC_50_: Half maximal inhibitory concentration
KuE: Lys-urea-Glu
LNCaP: Lymph node carcinoma of the prostate
PCa: Prostate cancer
PET: Positron emission tomography
PMPA: 2-Phosphonomethyl pentanedioic acid
PSMA: Prostate-specific membrane antigen
RP-HPLC: Reversed phase high-performance liquid chromatography
SCID: Severe combined immunodeficiency
SD: Standard deviation
SUV: Standardized uptake value
TFA: Trifluoroacetic acid
