# Supplementary material for: Synthesis and preclinical evaluation of novel 18F-labeled Glu-urea-Glu-based PSMA inhibitors for prostate cancer imaging: a comparison with 18F-DCFPyl and 18F-PSMA-1007
Source: EJNMMI Res. 2018 Apr 12;8:30. doi: 10.1186/s13550-018-0382-8 (PMC5897267; doi:10.1186/s13550-018-0382-8)
Supplement: Supplementary file 1 — Supporting information contains the description of the chemical synthesis and radiolabeling of all compounds investigated in this study, the methods and results for the determination of the PSMA binding affinities (IC50) and internalization studies, the metabolite analyses and the time-activity curves for the blood pool derived from dynamic small-animal PET. (PDF 911kb) [file 13550_2018_382_MOESM1_ESM.pdf]

Supporting Information

Synthesis and Preclinical Evaluation of novel ^{18}F-labeled Glu-urea-Glu-based PSMA inhibitors for Prostate Cancer Imaging: a comparison with ^{18}F-DCFPyl and ^{18}F-PSMA-1007

Stephanie Robu[1]*, Alexander Schmidt[1], Matthias Eiber[2], Margret Schottelius[1], Thomas Günther[1], Behrooz Hooshyar Yousefi[2], Markus Schwaiger[2] and Hans-Jürgen Wester[1]

[1] Chair of Pharmaceutical Radiochemistry, Technical University Munich, Walther-Meissner-Strasse 3, 85748 Garching, Germany

[2] Department of Nuclear Medicine, Klinikum rechts der Isar, Technical University Munich Ismaningerstr. 22, 81675 München, Germany

* to whom correspondence should be addressed

Stephanie Robu
Chair for Pharmaceutical Radiochemistry
Technical University Munich
Walther-Meissner-Str. 3
85748 Garching
Germany
Phone: +49 89 289 12203
Fax: +49 89 289 12204
stephanie.robu@tum.de

Chemical Synthesis

All protected amino acid analogs and the Tritylchloride polystyrene (TCP) resin (100-200 mesh; loading: 1.6 mmol/g) were purchased from Iris Biotech (Marktredwitz, Germany) or Bachem (Bubendorf, Switzerland). All other organic reagents and solvents were obtained from SigmaAldrich (Munich, Germany) or CLN (Freising, Germany). Solid phase peptide synthesis (SPPS) was carried out manually using an Intelli-Mixer syringe shaker (Neolab, Heidelberg). Analytical reversed-phase high performance liquid chromatography (RP-HPLC) was performed on a Nucleosil 100 C 18 (5µm, 125 x 4.0 mm) column (CS GmbH, Langerwehe, Germany) and semi-preparative RP-HPLC using a Multospher 100 RP 18-5 (250 x 20 mm) column (CS GmbH, Langerwehe, Germany). Analytical and preparative RP-HPLC was performed on a Sykam gradient HPLC System (Sykam GmbH, Fürstenfeldbruck, Germany). The peptides were eluted with 0.1 % TFA (v/v) in H_2O (Solvent A) and 0.1 % TFA in acetonitrile (Solvent B) with a constant flow of 1 mL/min for analytical RP-HPLC or 5 mL/min for preparative purification. The specific gradients, the retention times t_R as well as the capacity factors *K´* are cited in the text. For UV detection a 206 PHD UV-Vis detector (Linear™ Instruments Corporation, Reno, USA) was used. Radio-HPLC of the radioiodinated ligand and metabolic studies of the ^{18}F-labeled EuE-based inhibitors were performed on a Nucleosil 100 C 18 (5 µm, 125 x 4.0) column (flow: 2 mL/min)using a NaI(Tl) well-type scintillation counter from EG & G Ortect (München, Germany) for radioactivity detection (HPLC System A). Analytical Radio-HPLC of the ^{18}F-labeled derivatives and metabolic analyses of ^{18}F-PSMA-1007 was carried out using a Nucleosil 100 C 18 (5 µm, 125 x 4.0) column (flow: 1.5 mL/min) on a Shimadzu HPLC system equipped with a NaI(TI) scintillation detector (2" x 2") and a SPD M20A diode array UV/Vis detector (HPLC System B). ESI-MS spectra were obtained with a Varian 500-MS IT mass spectrometer (Agilent Technologies, Santa Clara, USA).

The synthesis of the EuE-based precursors for ^{18}F-labeling and the respective cold standards of the corresponding ^{18}F-labeled PSMA-inhibitors are summarized in Figure S1 A.

*Synthesis of EuE-k-Aoa (**1**) and EuE-k-ß-a (**2**):* The carboxyl-protected Glu-urea-Glu-core (O*t*Bu)EuE(O*t*Bu)$_2$ was synthesized as previously described [1]. The desired product was obtained as waxy solid in 84 % yield. HPLC (10% to 90% B in 15 min): t_R = 11.3 min; *K´* = 7.69. Calculated monoisotopic mass ($C_{23}H_{49}N_2O_9$): 488.3; found: *m/z* = 489.4 [M+H]$^+$, 511.4 [M+Na]$^+$.

Solid-phase peptide synthesis of EuE-k-Aoa and EuE-k-β-a was performed, using N-terminal-protected-amino acids or amino acid analogs (1.5 eq), HOBt (1.5 eq), TBTU (1.5 eq) and DIPEA (4.5 eq) in DMF according to a previously published protocol [1]. The final resin-bound peptide was deprotected and cleaved from the resin using Trifluoroacetic acid (TFA) for 1 h. The crude peptides were precipitated in diethyl ether and purified using RP-HPLC.

*EuE-k-Aoa (**1**)* (62% yield)*:* HPLC (10% to 50% B in 15 min): t_R = 5.32 min; *K´* = 3.09. Calculated monoisotopic mass ($C_{19}H_{31}N_5O_{12}$): 521.2; found: *m/z* = 522.3 [M+H]$^+$, 544.2 [M+Na]$^+$.

*EuE-k-ß-a (**2**)* (54% yield)*:* HPLC (0% to 15% B in 15 min): t_R = 7.90 min; *K´* = 5.08. Calculated monoisotopic mass ($C_{20}H_{33}N_5O_{11}$): 519.2; found: *m/z* = 520.2 [M+H]$^+$, 522.2 [M+Na]$^+$.

*Synthesis of EuE-k-FBOA (**3**):* Oxime ligation of EuE-k-Aoa (20.0 mg; 0.04 mmol; 1 eq.) with 4-fluorobenzaldehyde (5.7 mg; 0.05 mmol; 1.2 eq.) was carried out in MeCN/H_2O (1/1; acidified with TFA to pH 2.5) for 1 h at 60 °C. The final product was obtained in 87% yield after purified RP-HPLC purification. HPLC (15% to 30% B in 15 min): t_R = 13.2 min; *K´* = 9.15. Calculated monoisotopic mass ($C_{26}H_{34}FN_5O_{12}$): 627.3; found: *m/z* = 628.5 [M+H]$^+$, 650.5 [M+Na]$^+$.

*Synthesis of EuE-k-ß-a-FPyl (**4**):* 2,3,5,6-tetrafluorophenyl 6-fluoronicotinate was synthesized as published previously [2]. For the synthesis of EuE-k-ß-a-FPyl, 20 mg of EuE-k-ß-a (0.04 µmol, 1 eq.) were dissolved in DMF and 13.4 mg of 2,3,5,6-tetrafluorophenyl 6-fluoronicotinate (0.05 mmol, 1.2 eq) and 30.6 µL DIPEA (0.18 mmol, 4.5 eq.) were added. The reaction mixture was stirred for 1 h at RT. The final product was purified using RP-HPLC (82 % yield). HPLC (10% to 30% B in 15 min): t_R = 6.2 min; *K´* = 3.78. Calculated monoisotopic mass ($C_{26}H_{35}FN_6O_{12}$): 642.2; found: *m/z* = 643.3 [M+H]$^+$.

Synthesis of the DCFPyl-precursor and the cold reference compound DCFPyl: The Lys-urea-Glu precursor (O*t*Bu)KuE(O*t*Bu)$_2$ was synthesized as previously described and obtained in 94% yield [1]. Deprotection was carried out using TFA (1 h) and the crude product was precipitated in cold diethyl ether and purified using RP-HPLC (68 % yield): HPLC (0% to 10% B in 15 min): t_R = 2.2 min; K' = 1.17. Calculated monoisotopic mass ($C_{12}H_{21}N_3O_7$): 319.1; found: m/z = 320.1 $[M+H]^+$.

DCFPyl (75 % yield) was synthesized according to the protocol outlined for EuE-k-ß-a-FPyl (see above), starting from the DCFPyl-precursor (KuE) and 2,3,5,6-tetrafluorophenyl 6-fluoronicotinate: HPLC (0% to 40% B in 15 min): t_R = 5.9 min; K' = 3.54. Calculated monoisotopic mass ($C_{18}H_{23}FN_4O_8$): 442.2; found: m/z = 443.2 $[M+H]^+$.

Synthesis of the PSMA-1007 precursor and the cold standard PSMA-1007: The precursor for PSMA-1007 was synthesized in accordance with the protocol described for ^{177}Lu-DKFZ-617 [3] and was obtained in 63 % yield: HPLC (20% to 40% B in 15 min): t_R = 8.9 min; K' = 5.84. Calculated monoisotopic mass ($C_{43}H_{59}N_7O_{15}$): 913.4; found: m/z = 914.5 $[M+H]^+$.

The conjugation of 2,3,5,6-tetrafluorophenyl 6-fluoronicotinate to the PSMA-1007 precursor was performed in analogy to the synthesis of EuE-k-β-a-FPyl**.** PSMA-1007 (cold reference) was obtained in 87 % yield: HPLC (20% to 50% B in 15 min): t_R = 9.1 min; K' = 6.00. Calculated monoisotopic mass ($C_{49}H_{61}FN_8O_{16}$): 1036.4; found: m/z = 1037.3 $[M+H]^+$.

*Synthesis of N,N,N-Trimethyl-5-((2,3,5,6-tetrafluorophenoxy)carbonyl)-pyridin-2-aminium Trifluoromethanesulfonate (**5**):* The synthesis was carried out as previously described [4].
HPLC (10% to 90% B in 15 min): t_R = 8.5 min; K' = 5.54. Calculated monoisotopic mass ($C_{15}H_{13}F_4N_2O_2^+$): 329.0; found: m/z = 329.1 $[M+H]^+$.

*Synthesis of 4-Formyl-N,N,N-anilinium perchlorate (**6**):* The synthesis was performed as previously described. HPLC (10% to 30% B in 15 min): t_R = 3.47 min; K' = 1.67. Calculated monoisotopic mass ($C_{10}H_{14}NO^+$): 164.1; found: m/z = 164.2 $[M+H]^+$[4].

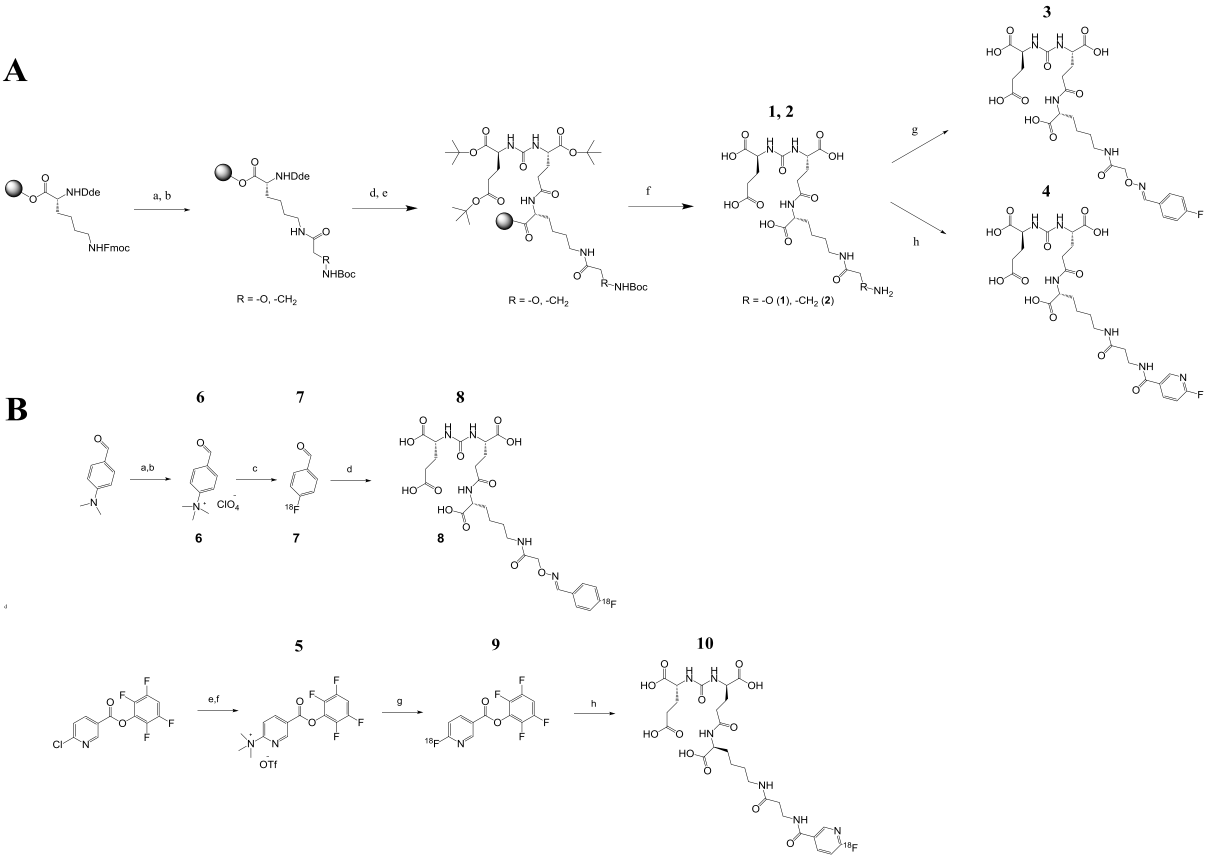

Figure S1 **A) Synthesis of the EuE-based precursors (1, 2) and the cold reference standards of the ^{18}F-labeled derivatives (3, 4)**

(a) 20% piperidine in NMP; **(b)** Boc-AoAc-OH, HOBt, TBTU, DIPEA [DMF]/ Boc-beta-Ala-OH, HOBt, TBTU, DIPEA [DMF]; **(d)** 2% hydrazine in DMF; **(e)** (O*t*Bu)EuE(O*t*Bu)$_2$, HOBt, TBTU, DIPEA [DMF]; **(f)** TFA; **(g)** 4-fluorobenzaldehyde [MeCN/water; 1/1, (v/v), acidified with TFA to pH 2.5]; **(h)** 2,3,5,6-tetrafluorophenyl 6-fluoronicotinate**,** DIPEA, [DMF]

B) ^{18}F-labeling of the prosthetic groups (5, 6) and synthesis of the ^{18}F-labeled EuE-based PSMA-inhibitors EuE-k-^{18}F-FBOA (8) and EuE-k-ß-a-^{18}F-FPyl (10)

(a) 4-(dimethylamino)benzaldehyde, CH_3I [acetone]; **(b)** $AgClO_4$ [DMF]; **(c)** $^{18}F^-$, 150°C [DMSO]; **(d) 1,** [MeCN/water/TFA; 2/1, (v/v), pH 2.5]; **(e)** Me_3N [THF]; **(f)** TMSOTf [DCM]; **(g)** $^{18}F^-$, 55°C [EtOH/MeCN/*tert*-butanol; 1/3/1, (v/v/v)]; **(h) 2**, $NaHCO_3$ [MeCN/water; 2/1, (v/v)]

Radiolabeling

The ^{18}F-labeling of the prosthetic groups ^{18}F-FBA (**7**) and ^{18}F-FPyl-TFP (**9**) and the conjugation of the respective EuE-based precursor are shown in Figure S1 B.

*Synthesis of 4-^{18}F-fluorobenzaldehyde (^{18}F-FBA) (**7**):* The ^{18}F-labeling of 4-formyl-*N,N,N*-trimezhylbenzenaminium perchlorate was performed in accordance to a previously published protocol with minor modifications [4]. Briefly, $^{18}F^{-}$ was eluted from a QMA cartridge with 10 mg precursor in MeOH. The solvent was evaporated under argon flow at 70–80 °C and the residue was dissolved in DMSO. The resulting solution was heated at 150 °C for 10 min, ^{18}F-FBA was loaded on a polymeric RP-cartridge (Strata-X, 30 mg) and eluted using MeCN.

*Synthesis of EuE-k-^{18}F-FBOA (**8**):* ^{18}F-FBA (1200 - 3500 MBq) in 300 µL of MeCN was added to 1.7 mg of EuE-k-AoA (3.26 µmol) dissolved in water/MeCN (1/3, acidified with TFA to pH 2.5). Oxime ligation was carried out for 15 min at 60 °C. After diluting with 1 mL water, the final product was purified using RP-HPLC. The product fraction was diluted with 10 mL water and loaded onto a C18 Sep Pak Plus cartridge, which had been preconditioned with 3 mL EtOH and rinsed with 10 mL water. After immobilization of the ^{18}F-labeled product, the cartridge was washed with 5 mL water, and the final product was eluted with EtOH and diluted with phosphate-buffered saline (pH 7.4) for further studies. HPLC (5% to 35% B in 15 min): t_R = 12.3 min; *K´* = 8.46.

*Synthesis of 2,3,5,6-tetrafluorophenyl 6-^{18}F-fluoronicotinate (^{18}F-FPyl-TFP) (**9**):* The ^{18}F-labeling of *N,N,N*-5-[(2,3,5,6-tetrafluorophenoxy)-carbonyl]pyridine-2-aminium triflouromethanesulfonate was performed in accordance to a previously published protocol [5]. Briefly, $^{18}F^{-}$ was eluted from a QMA cartridge with 13 mg precursor in EtOH and 2 ml MeCN/*t*BuOH (1/4, (v/v)) were passed through the cartridge into the reaction vessel. The resulting solution was heated at 55 °C for 15 min, ^{18}F-FPyl was loaded on a polymeric RP-cartridge (Strata-X, 60 mg) and eluted using MeCN.

*Synthesis of EuE-k-ß-a-^{18}F-FPyl (**10**), ^{18}F-DCFPyl and ^{18}F-PSMA-1007:* Conjugation of *^{18}F-FPyl-TFP* with the respective precursor was performed as previously published [6]. ^{18}F-FPyl-TFP in MeCN was added to a vial containing the peptide precursor (3.9 µmol) and 5.5 mg $NaHCO_3$ dissolved in water. The coupling reaction was carried out at 60 °C for 15 min. The final products were purified by RP-HPLC, followed by SPE extraction in analogy to the synthesis of *EuE-k-^{18}F-FBOA*.

*EuE-k-ß-a-^{18}F-FPyl (**10**):* HPLC (5% to 35% B in 15 min): t_R = 6.8 min; K' = 4.23.

^{18}F-DCFPyl: HPLC (5% to 5% B in 15 min): t_R = 4.3 min; K' = 2.30.

^{18}F-PSMA-1007: HPLC (5% to 95% B in 15 min): t_R = 7.2 min; K' = 4.53.

The radioiodinated reference ligand ([^{125}I]I-BA)KuE ((S)-1-carboxy-5-(4-(-^{125}I-iodo-benzamido)pentyl)carbamoyl)-L-glutamic acid) was prepared as previously described [1].

Determination of the PSMA binding affinities (IC_{50})

The culture medium was removed and the cells were washed with 500 µL of HBSS (Hank´s balanced salt solution, Biochrom Berlin, Germany, containing 1% bovine serum albumin (BSA)). Before the experiment the cells were left to equilibrate in 200 µL HBSS (1% BSA) for 15 min on ice. Afterwards, 25 µL/well of solutions containing the respective unlabeled inhibitor in increasing concentrations (10^{-10} – 10^{-4} M in HBSS (1% BSA)) or either HBSS (1% BSA) as control were added, followed by the addition of 25 µL of ([^{125}I]I-BA)KuE as a competitor. The final concentration of the radioiodinated inhibitor was 0.2 nM in all assays. For each concentration the experiments were performed in triplicates. Incubation of the cells was carried out for 60 min on ice and terminated by removal of the assay medium. The cells were thoroughly rinsed with 250 µL of HBSS and the washing medium was combined with the supernatant of the previous step, representing the amount of free radioligand. Then, the cells were lysed using 250 µL of 1 N NaOH, and the lysat was transferred to vials and combined with 250 µL of HBSS, which was used for an additional washing step (bound activity). The Quantification of the amount of free and bound radioactivity was performed in a γ-counter. IC_{50} curves of the respective PSMA inhibitors are shown in Fig S2.

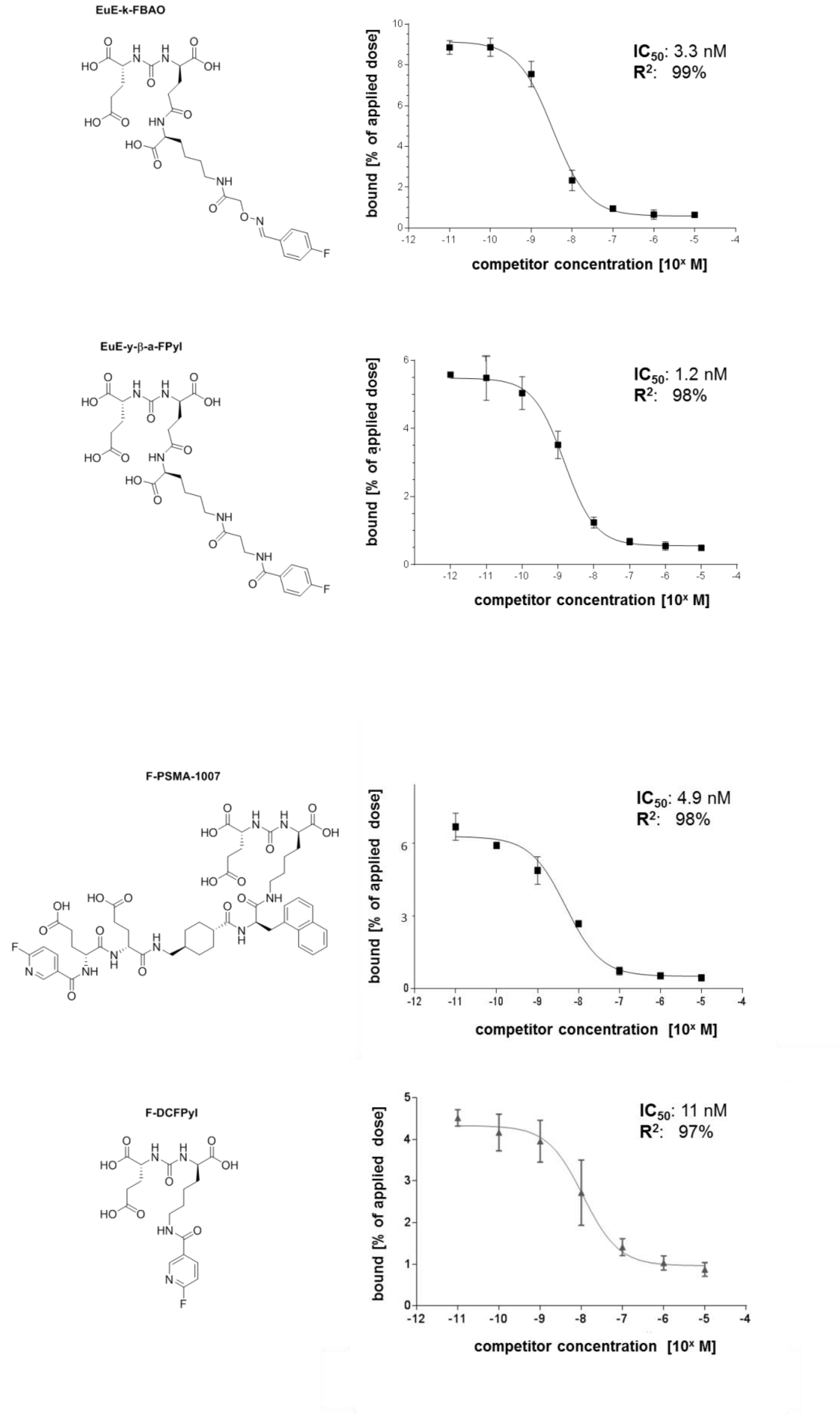

Fig S2 IC_{50} curves of the cold EuE-based inhibitors, F-DCFPyl and F-PSMA-1007. Binding assays were performed using LNCaP cells and ([^{125}I]I-BA)KuE (0.2 nM) as radioligand (1 h, 4°C)

Internalization studies

Internalization studies of the ^{18}F-labeled EuE-based inhibitors, ^{18}F-DCFPyl and ^{18}F-PSMA-1007 were performed according to a previously published protocol [1]. Briefly, after washing the cells with 0.5 mL DMEM-F12 (5% BSA), the cells were preconditioned for a minimum of 15 min with 200 µL DMEM-F12 (5% BSA) at 37°C. Then, 25 µL (per well) of either assay medium (total internalization) or of a 100 µM 2-PMPA solution (non-specific internalization) were added, followed by addition of 25 µL of assay medium containing the respective ^{18}F-labeled PSMA inhibitor or ([^{125}I]I-BA)KuE (0.2 nM). The internalization kinetics were determined by cell incubation at 37°C for 5, 15, 30, and 60 min, respectively. All experiments were carried out in triplicate for each time point (control and blocking). Incubation was terminated by placing the plate on ice and by removal of the incubation medium. The cells were subsequently rinsed with 250 µL DMEM-F12 (5% BSA) and combined with the supernatant of the previous step (amount of free radioligand). To remove receptor surface-bound radioactivity, the cells were incubated with 250 µL of ice cold PMPA in PBS (10 µM) for 10 min. After removal of the supernatant, the cells were thoroughly rinsed with another 250 µL of ice-cold PBS and both fractions were combined (amount of receptor-bound radioactivity). By incubation of the cells with 250 µL 1N NaOH (5 min), the internalized activity was released, the suspension transferred to vials, and combined with 250 µL of 1N NaOH used for subsequent rinsing of the wells. Quantification of the amount of free, PMPA-releasable, and internalized activity was performed in a γ-counter. For analysis of the internalization, data for both the ^{18}F-labeled compound of interest and for the reference ([^{125}I]I-BA)KuE in the same experiment were first corrected by the amount of non-specific internalization (adding of 100 µM PMPA), respectively, and then each was normalized to the amount of internalized ligand in the absence of unlabeled competitor (100%). To eliminate the influence of cell count and cell viability on the absolute internalization values, data (60 min) are expressed as the ratio of the internalization of the compound of interest to the internalization (60 min) found for ([^{125}I]I-BA)KuE in the same experiment (Fig 2, Fig S3). Data represent means ± SD (n=3).

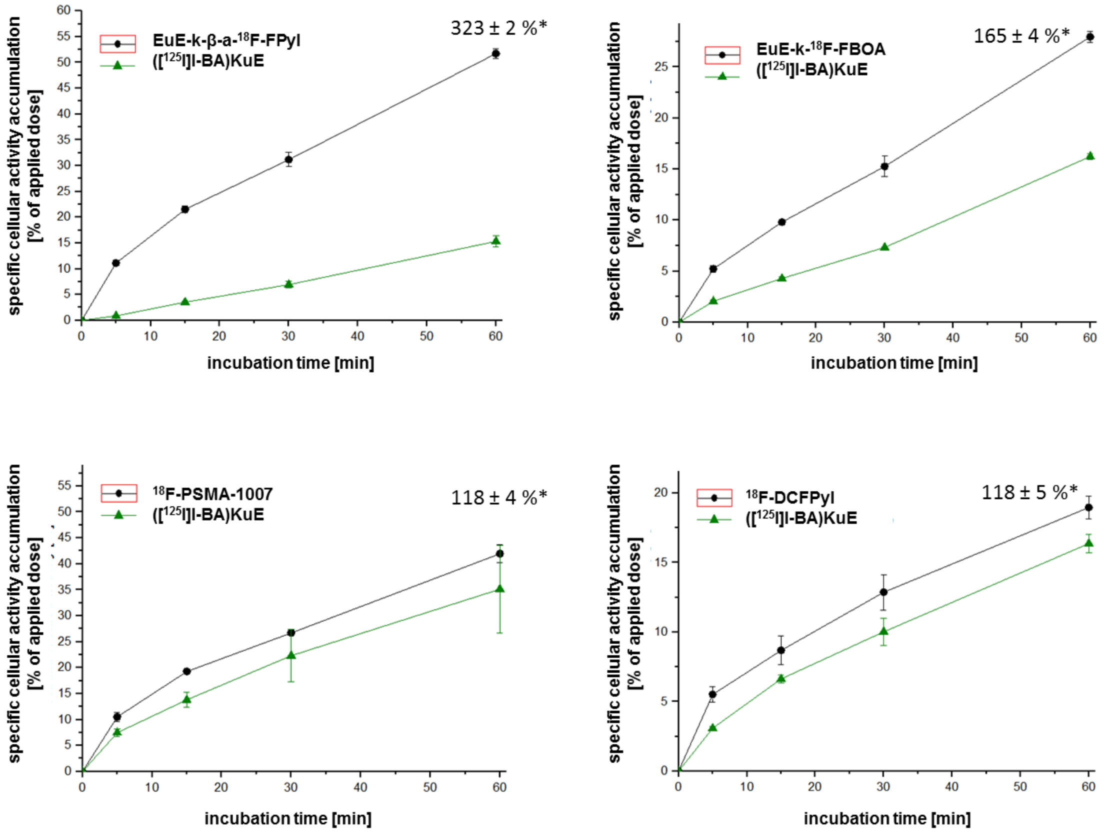

Fig S3 Kinetics of PSMA-mediated internalization of EuE-k-^{18}F-FBOA, EuE-k-β-a-^{18}F-FPyl, ^{18}F-DCFPyl, ^{18}F-PSMA-1007 and reference ([^{125}I]I-BA)KuE into LNCaP cells (37°C) (mean ± SD [n = 3])

*Specific internalization of the reference compound ([^{125}I]I-BA)KuE assayed in a parallel experiment and used for data normalization

Metabolite Analysis

The results of the metabolite analyses of the ^{18}F-labeled EuE-based inhibitors, as well as of ^{18}F-PSMA-1007 are summarized in Fig S4.

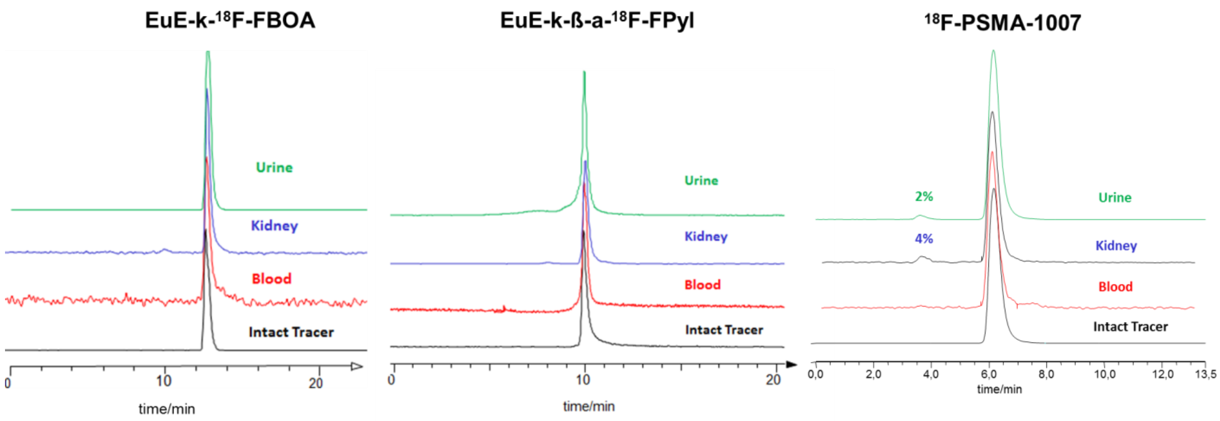

Fig S4 *In vivo* stability of the ^{18}F-labeled EuE-based inhibitors EuE-k-^{18}F-FBOA, EuE-k-β-a-^{18}F-FPyl and ^{18}F-PSMA-1007 in CD-1 nu/nu mice. Curves represent RP-HPLC chromatograms of intact tracer (quality control), blood, urine and kidney homogenate samples collected 1h p.i. of the respective radioligands

Small-animal PET studies

A comparison of the blood pool clearance of EuE-k-^{18}F-FBOA, EuE-k-β-a-^{18}F-FPyl, ^{18}F-DCFPyl and ^{18}F-PSMA-1007 in CD-1 nu/nu mice derived from dynamic small animal PET data is shown in Fig S5.

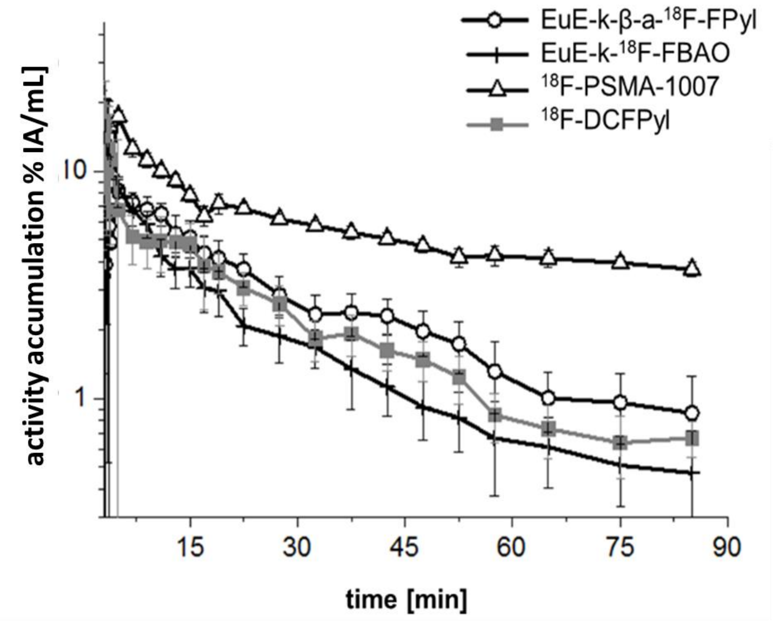

Fig S5 Time-activity curves (logarithmic plot) in % IA/mL for the blood pool (heart) derived from dynamic small animal PET

References

1. Weineisen, M., et al., *Synthesis and preclinical evaluation of DOTAGA-conjugated PSMA ligands for functional imaging and endoradiotherapy of prostate cancer.* EJNMMI Res, 2014. **4**(1): p. 014-0063.
2. Yue, X., et al., *One-pot two-step radiosynthesis of a new (18)F-labeled thiol reactive prosthetic group and its conjugate for insulinoma imaging.* Mol Pharm, 2014. **11**(11): p. 3875-84.
3. Benesova, M., et al., *PSMA-617 - a novel theranostic PSMA inhibitor for both diagnosis and endoradiotherapy of prostate cancer.* Journal of Nuclear Medicine, 2015. **56**(supplement 3): p. 63.
4. Richarz, R., et al., *Neither azeotropic drying, nor base nor other additives: a minimalist approach to (18)F-labeling.* Org Biomol Chem, 2014. **12**(40): p. 8094-9.
5. NEUMAIER, B.Z., Boris; RICHARZ, Raphael; KRAPF, Phillip;, *METHOD FOR THE PRODUCTION OF 18F-LABELED ACTIVE ESTERS AND THEIR APPLICATION EXEMPLIFIED BY THE PREPARATION OF A PSMA-SPECIFIC PET-TRACER*. 2016, MAX-PLANCK-GESELLSCHAFT ZUR FÖRDERUNG DER WISSENSCHAFTEN E.V.
6. Malik, N., et al., *Radiosynthesis of a new PSMA targeting ligand ([18F]FPy-DUPA-Pep).* Appl Radiat Isot, 2011. **69**(7): p. 1014-8.
